# Criminological characteristics of sexual assault perpetrators: a Tunisian study

**DOI:** 10.1192/j.eurpsy.2023.1862

**Published:** 2023-07-19

**Authors:** A. Chamseddine, N. Charfi, O. Bouattour, R. Feki, I. Gassara, N. Smaoui, S. Omri, L. Zouari, J. Ben Thabet, M. Maâlej Bouali, M. Maâlej

**Affiliations:** Psychiatry C, Hedi Chaker Hospital in Sfax, Sfax, Tunisia

## Abstract

**Introduction:**

In Tunisia, Sexual assaults constitute a pervasive problem that concerns the health care system and the country’s judicial authorities alike.

**Objectives:**

The aims of our study were to estimate the incidence of sexual assault encountered in the context of forensic psychiatric assessment and to assess the criminological profile of sexual assault perpetrators.

**Methods:**

We conducted a retrospective study of a series of sexual assault perpetrators examined in a forensic psychiatric assessment in the psychiatry C department at Hedi Chaker university Hospital in Sfax, from January 2010 to December 2021.

**Results:**

Over the period of 11 years (2010 to 2021), we collected 374 forensic psychiatric assessment files. Among them, 49 were those of sexual assaults (13.10%). It was a rape assault in 54.4% of cases. Aggravating circumstances have been noted in 87% of cases. These were mainly assaults on minors (54.3%) and assaults associated with physical violence (38.4%).

The victim gender was female in 63% of cases, with an average age of 21 years 9 months. and was among the relatives of the sexual assault perpetrator in 28.3% of cases.

**Conclusions:**

Rape seems to represent a non-negligible proportion of the offenses motivating a forensic psychiatric assessment in Tunisia. Sexual assaults against minors are frequent, despite undeniable under-reporting.

**Disclosure of Interest:**

None Declared

